# Minimally invasive technique combined with external fixator in the treatment of pediatric flexion-type humeral supracondylar fractures

**DOI:** 10.1038/s41598-023-48158-6

**Published:** 2023-12-14

**Authors:** ChengMing Zhu, QiYuan Feng, ZiXuan Ou, HaoBo Zhong, Xin Tang

**Affiliations:** 1 Department of Orthopaedic, Liuzhou Workers Hospital/the Fourth Affiliated Hospital of Guangxi Medical University, Liuzhou, 545007 China; 2 Tongji Medical College, Huazhong University of Science and Technology, Wuhan, 430030 China; 3 Union Hospital, Tongji Medical College, Huazhong University of Science and Technology, Wuhan, 430030 China; 4 Department of Orthopaedics, Huizhou First Hospital, Huizhou, 516000 China

**Keywords:** Trauma, Bone

## Abstract

Flexion-type pediatric humeral supracondylar fractures are rare, and the reduction technique remains contradictory. A minimally invasive technique using percutaneous leverage reduction combined with an external fixator was described to achieve satisfactory reduction and avoid the open reduction in this study. The operation and clinical results of patients treated with this technique were retrospectively compared with traditional closed reduction. From January 2013 to January 2018, children diagnosed with displaced flexion-type humeral supracondylar fractures were included in this study. Patients were treated with closed reduction (Group A) or minimally invasive reduction technique (Group B). The external fixator fixation was then applied. The demographic information, as well as the clinical and functional results of the operation, were retrospectively reviewed and evaluated. There were twenty-two patients, ten in Group A and twelve in Group B. The mean duration of the operation in Group A was more prolonged than Group B (59 min versus 46 min, *p* < 0.001). No infection, nonunion, myositis ossificans, neurovascular injury or other complications related to the operation were observed by the time the fractures healed. During an average 36 months follow-up time, almost all children achieved good to excellent results except for one fair in Group A according to the MEPS and the Flynn criteria. This study introduced a safe and efficient minimally invasive technique for displaced flexion-type supracondylar humerus fractures. With the assistance of mosquito forceps, this leverage technique might achieve similar satisfactory clinical outcomes as traditional closed reduction but with a shorter surgical duration.

## Introduction

Supracondylar humeral fracture (SHF) is the most frequently occurring injury in pediatric orthopaedics, which constitutes 50–70% of all elbow fractures in children, associated with a high rate of neurological complications^1,2^. Among children's humeral supracondylar fractures, the extension type shares the highest proportion, while the flexion type only accounts for 2–5%^3,4^. Flexion-type SHF typically occurs when there is a direct impact to the back of the elbow, causing a reverse angulation of displacement compared to extension-type SHF^5,6^. This type of fracture is a rare clinical situation and is seldom reported in the literature.

Unlike extension-type SHF, the risk of nerves and blood vessel damage is lower in flexion-type SHF. But the reduction and fixation remain challenging to pediatric orthopaedic surgeons. ^3,7^. The normalized guidelines by the American Academy of Orthopedic Surgeons (AAOS) currently recommend closed reduction with percutaneous pinning fixation for displaced SHF^8^. However, in severely displaced flexion-type supracondylar fractures, the distal fracture fragment might move forward to damage the ulnar nerve, and the proximal end of the fracture might embed in the triceps brachii^9,10^. It might be tough to achieve sufficient reduction by traditional technique in this situation, and the open approach had to be performed in case of repeated failure of closed reduction. But this method might bring extensive surgical injury and increase the chances of infection and nonunion^11,12^. On the other hand, SHF is highly unstable, which poses a challenge in stabilizing the fracture fragments during percutaneous pinning fixation^12,13^.

In order to address these issues, this study described a minimally invasive reduction technique combined with the external fixator to achieve satisfactory reduction and avoid open reduction. This technique was compared with the traditional closed reduction in the treatment of displaced flexion-type supracondylar humeral fractures.

## Patients and methods

From January 2013 to January 2018, children under age 14 with a diagnosis of displaced flexion-type SHF treated in the corresponding author’s institute were retrospectively reviewed. The inclusion criteria required patients who had been followed up for over two years with complete clinical and radiological data. Patients diagnosed with concomitant neurovascular injury needing open exploration surgery or metabolic bone disease were excluded from this study. All the patients got treatment with traditional closed reduction (Group A) or minimally invasive reduction (Group B). The demographic information, including age, gender, operation duration and follow-up time of the two groups, were listed in Table 1. Detailed information on the surgical procedure was provided to all parents or guardians of the patients, and all of them gave consent to be involved in the study. The corresponding authors' ethical review committee provided the approval for this research. All methods were performed in accordance with the relevant guidelines and regulations.

**Table 1:** Demographics of patients.

	Group A (n = 10) Mean ± SD	Group B (n = 12) Mean ± SD	*P*
Age (years)	8.7 ± 1.8	9.5 ± 2.1	0.359
Gender (male/female)	10/0	6/6	0.009
Operation duration (minutes)	59.5 ± 5.1	46.6 ± 3.0	< 0.001
Follow-up (months)	39.0 (15.0) *	34.8 ± 9.3	0.346

SD, standard deviation.

*Non-normal distribution, Mean (interquartile range).

### Surgical technique

**Figure 1:**
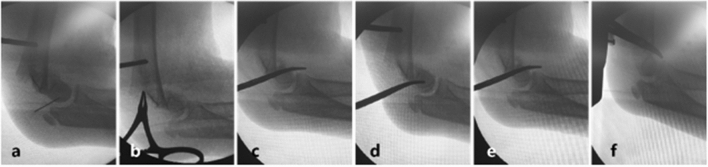
Surgical procedure of the minimally invasive reduction technique. (**a**) Make a small incision from the posterior side of the elbow; (**b**) Insert the mosquito forceps to help release the interposed soft tissue between fractures; (**c**–**f**) Pull the distal fragment and achieve reduction using lever technique.

Patients were placed supine under general anesthesia, and all surgery was performed by the same surgeons’ team. The closed reduction technique was performed for patients of Group A. The coronal rotation deformity was correct firstly, overlapping and mediolateral translation through axial traction under the guidance of C-arm fluoroscopy. Then longitudinal traction was applied while the elbow was extend to restore the sagittal displacement, which is different from extension type supracondylar fractures fromf f^4^. This procedure would be repeated until the fracture reached a satisfying reduction. In Group B, all the patients could not achieve an acceptable reduction after two attempts of close reduction under fluoroscopy. Therefore, the minimally invasive assistance reduction technique was performed. A small incision was made on the fracture level from the posterior side of the elbow. A mosquito forceps was inserted to separate the tissue from the skin surface. The tip of the mosquito forceps could be inserted between the fracture fragments to release the interposed soft tissue (Fig. 1). The distal fragment was pulled and gradually achieved reduction using the lever technique^14^.

**Figure 2:**
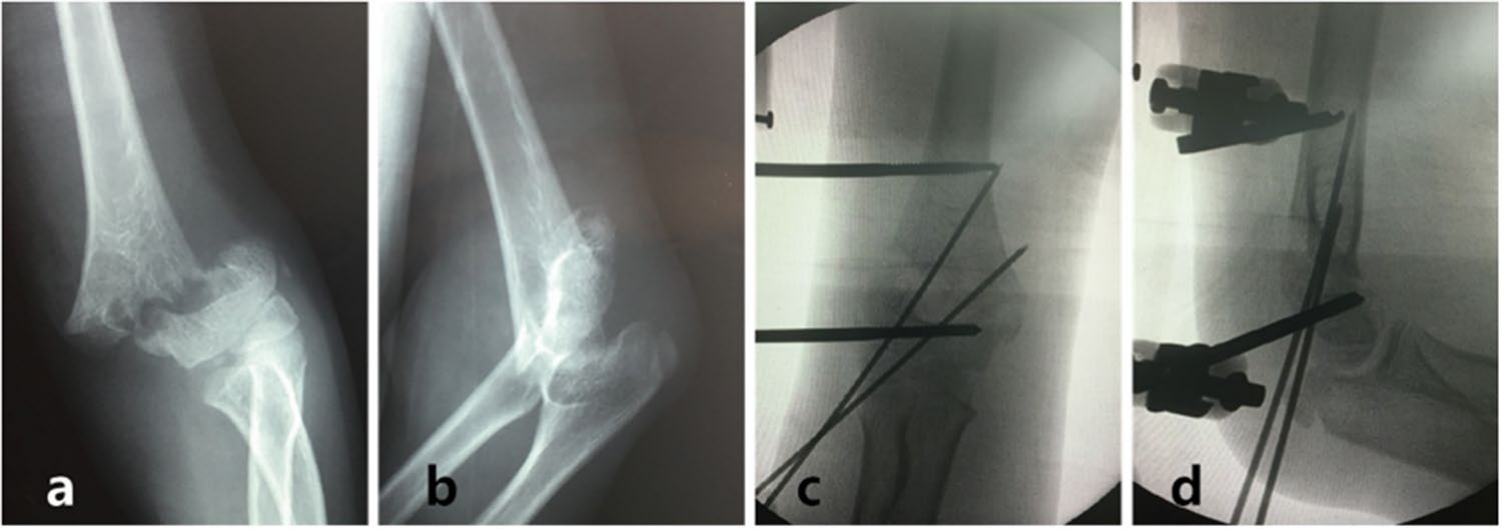
A 13-year-old boy diagnosed with flexion-type SHF of his left elbow. (**a**, **b**) The anteroposterior (AP) and lateral radiographic images on admission; (**c**, **d**) The post-operative AP and lateral radiographs.

The proximal Schanz pin could be first inserted to ensure the stability of the fracture fragments at 90° to the longitudinal axis of the humerus. This Schanz pin is inserted 2 cm proximal to the fracture line and avoiding injury to the radial nerves, with a mosquito forceps separating the soft tissue and nerves before the Schanz pin is inserted. Once satisfied reduction was achieved, the distal pin was inserted at 1.0 cm from the epiphyseal plate and parallel to the epiphyseal line. The second pin should not be fully perforating the medial cortex of the distal part of the humerus in order to avoid injury to the ulnar nerve. The Schanz pin clamps were then tightened, and percutaneous K-wire fixation was carried out under the guidance of the C-arm using two 1.5 to 2.0 mm K-wires^15,16^. Fluoroscopic pictures were taken to validate the reduction quality (Fig. 2).

### Postoperative care and follow-up

The blood circulation and sensation should be checked for 1–2 days after the operation. Then the patients were discharged without a cast, and free range of movement without weight-bearing of the elbow was permitted. The elbow joint functional exercise was gradually advanced. The patients returned for clinical evaluations at 3, 6, 12 weeks and 6, 12, and 24 months postoperatively. Regular X-ray examination was performed at 3, 6, 12 weeks and 6, 12 months after surgery. The external fixation pins and K-wires were removed 6 to 8 weeks after surgery, when radiographic outcomes proved fracture healing. The recovery of elbow function was evaluated according to the criteria of the MEPS^17^ and Flynn^18^ at the last follow-up.

### Statistical analysis

Statistical analysis was performed using the IBM SPSS statistics version 20 software (SPSS Inc., Chicago, Illinois). The Student t-test and Mann–Whitney U test were used for descriptive statistics and comparative analysis between two groups.

### Ethical approval and consent to participate

The Ethics Committee of Tongji Medical College, Huazhong University of Science and Technology (IORG No: IORG0003571) gave a final APPROVAL for this study. Although the data were collected anonymized and centrally, all guardians of patients signed written informed consent for participate.

## Results

This study included six girls and sixteen boys, with a mean age of nine years. Ten patients were in Group A (close reduction), and others were in Group B (minimally invasive reduction). The two groups were similar in age and follow-up period. No infection, nonunion, myositis ossificans, neurovascular injury or other complications related to the operation were observed. The operation time in Group A was longer than in Group B, with statistical significance (*P* < 0.001) (Table 1).

The carrying angles of fractured and uninjured sides were measured at the last follow-up, and the difference between the two sides was calculated. The results revealed that Group B’s carrying angle loss was significantly smaller than Group A’s (P < 0.05). The cosmetic recovery results in both groups were considered acceptable based on the Flynn criteria (< 10°). According to the MEPS and Flynn criteria, patients evaluated as having excellent and good elbow joint function reached 100% in Group B. However, one patient in Group A scored 85 on the MEPS criterion and fair on the Flynn criterion, with a motion loss large than 10° (Table 2).

**Table 2:** Flynn and MEPS outcome during the last follow-up.

Group	Functional results of elbow motion						Cosmetic results
	Flynn			MEPS			Carrying angle difference between fractured and uninjured sides
	Excellent	Good	Fair	Excellent	Good	Fair	
A (n = 10)	5	4	1	10	0	0	3.00 (1.75) *
B (n = 12)	7	5	0	12	0	0	1.92 ± 1.31^#^
							p = 0.025

*Non-normal distribution, Mean (interquartile range).

^#^Mean ± SD. SD, standard deviation.

## Discussion

Flexion-type supracondylar humerus fractures account for approximately 2% of all humeral fractures in children and primarily arise from a direct crush of the elbows in flexion position^19^. The distal fracture end is displaced anteriorly and medially, which might crush the ulnar nerve and leave the posterior side of the elbow bruised in severely displaced cases^20^. Achieving and maintaining a satisfactory reduction is challenging when treating this type of supracondylar fracture.

Closed reduction with percutaneous K-wire fixation is currently the standard treatment for supracondylar humerus fractures^21^. However, this technique still shows limitations. Closed reduction is difficult in patients with displaced fractures because of severe oedema and soft tissue interposition, which might lead to nerve or muscle injury after repeated manipulations. Open reduction is recommended under this condition, but patients’ parents tend to complain about the cosmetic appearance and longer hospital stays. The disruption of blood supply around the fracture end is also a concern^22,23^. Moreover, the risk of fracture fragment displacement is high during the insertion of K-wires. The incidence of associated complications is more probably to occur, including loss of elbow range of motion, cubitus varus and elbow stiffness^24,25^. And repeated adjustments were usually required to ensure the accuracy of the K-wires entry under fluoroscopy, increasing the operation duration time and the radiation exposure for patients and medical staff^26^.

To compensate for the deficiency of closed reduction, Lee et al. introduced the technique named pin leverage to help fracture reduction in 2007^27^. Lin et al. updated the technique by using mosquito forceps instead of a blunt pin, which could dissect the soft tissues more easily and lower the damage to neurovascular bundles^13^. The following literature gave good clinical and radiological outcomes, which showed that minimally invasive percutaneous leverage reduction could be a safe alternative in the treatment of humeral supracondylar fracture^28–30^. However, no literature reported this technique on the reduction of pediatric flexion-type SHF. In this study, the mosquito forceps were inserted from the posterior side of the elbow to avoid important nerves and blood vessels and then moved forward to release the hematoma around the fracture site. This technique made it possible to achieve satisfactory reduction without the need for open surgery. At the last follow-up, patients in both groups received comparable outcomes according to the Flynn and MEPS criteria.

Furthermore, the maintenance of the reduction is the other urgent problem to be solved. During the percutaneous pinning fixation for unstable fractures, it is difficult to avoid re-displacement of the reduced fragments because of the stress from drilling pins^31,32^. But the introduction of an external fixator used in this series provided an easy and fast way of rigid stabilization, which avoids the repeated insertion of K-wires. The surgery duration is significantly shortened, and the radiation exposure for patients and medical staff could be decreased. With the stability provided by the external fixator, the patients were allowed to start active mobilization of the elbow postoperatively. This fixation technique enabled early function exercise and contributed to the recovery of elbow joint function.

To the best of our knowledge, this is the first comparative study of traditional closed reduction and minimally invasive reduction technique for pediatric flexion-type SHF. The minimally invasive technical strategies was optimized and simplified with detail. The comparable outcomes with traditional methods, less operation time and carrying angle loss in the treatment of flexion-type SHF are the strengths of this technique.

There were several limitations in this study. The small sample size limits statistical power in this study, likely due to the relatively low incidence of flexion-type SHF. The sequential selection bias of study cases was another limitation of this study. The patients treated with closed reduction might be more complicated than those treated with the minimally invasive technique. Prospective, randomized studies with large samples in the future are necessary to validate this technique further.

## Conclusion

The minimally invasive technique described in our study appears to be a safe and efficient procedure for the treatment of pediatric flexion-type SHF. Compared with the traditional closed reduction procedure, this technique could achieve similar satisfactory clinical outcomes with shorter surgical duration.

## Data Availability

The datasets used and/or analysed during the current study are available from the corresponding author on reasonable request.

## Abbreviation

SHF: Supracondylar humeral fracture
